# Eco-evolutionary dynamics of pathogen immune-escape: deriving a population-level phylodynamic curve

**DOI:** 10.1098/rsif.2024.0675

**Published:** 2025-04-02

**Authors:** Bjarke Frost Nielsen, Chadi M. Saad-Roy, C. Jessica E. Metcalf, Cécile Viboud, Bryan T. Grenfell

**Affiliations:** ^1^ High Meadows Environmental Institute, Princeton University, Princeton, NJ, USA; ^2^ Miller Institute for Basic Research in Science, University of California, Berkeley, CA, USA; ^3^ Department of Integrative Biology, University of California, Berkeley, CA, USA; ^4^ Department of Ecology and Evolutionary Biology, Princeton University, Princeton, NJ, USA; ^5^ Division of International Epidemiology and Population Studies, Fogarty International Center, National Institutes of Health, Bethesda, MD, USA

**Keywords:** pathogen evolution, immune evasion, mathematical model, phylodynamics

## Abstract

The phylodynamic curve (Grenfell *et al*. 2004 *Science* **303**, 327–332 (doi:10.1126/science.1090727)) conceptualizes how immunity shapes the rate of viral adaptation in a non-monotonic fashion, through its opposing effects on viral abundance and the strength of selection. However, concrete and quantitative model realizations of this influential concept are rare. Here, we present an analytic, stochastic framework in which a population-scale phylodynamic curve emerges dynamically, allowing us to address questions regarding the risk and timing of the emergence of viral immune escape variants. We explore how pathogen- and population-specific parameters such as strength of immunity, transmissibility, seasonality and antigenic constraints affect the emergence risk. For pathogens exhibiting pronounced seasonality, we find that the timing of likely immune-escape variant emergence depends on the level of case importation between regions. Motivated by the COVID-19 pandemic, we probe the likely effects of non-pharmaceutical interventions (NPIs), and the lifting thereof, on the risk of viral escape variant emergence. Looking ahead, the framework has the potential to become a useful tool for probing how natural immunity, as well as choices in vaccine design and distribution and the implementation of NPIs, affect the evolution of common viral pathogens.

## 1. Introduction

The emergence of novel viral variants is widely understood to be a highly stochastic phenomenon [1,2 ] and it follows that any statements concerning viral adaptation must be statistical in nature. This does not, however, detract from their usefulness or fundamental importance: the ability to pinpoint risk factors for the emergence of, for example, an immune escape variant is of great value to public health, especially if quantitative.

**Figure 1.**
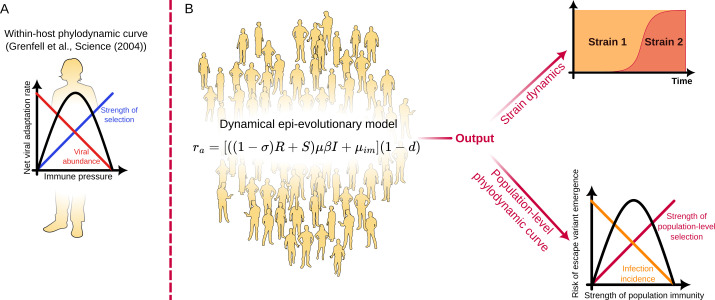
The model framework. (A) The within-host phylodynamic curve, a concept introduced by Grenfell *et al*. [3]. When the immune response is sufficiently strong, the abundance of virions is low and this limits viral adaptation. If the immune response is very weak or absent, there is little selection for immune-evasion, and thus adaptation is again limited. Peak adaptation is observed at intermediate strength of the immune response. (B) A dynamical population-scale model is introduced, relying only on a few key assumptions about imperfect immunity, transmission and the occasional appearance of immune-evading mutations in infected hosts. From this framework, a population-scale analogue of the phylodynamic curve dynamically emerges, addressing the risk of an immune escape variant arising and establishing in the population. Furthermore, the model allows exploration of the time-varying risk of immune escape in dynamical epidemiological settings. ProPublica's *Wee People* font is used for human silhouettes.

One example of such a statement is the phylodynamic curve (see figure 1A), introduced by Grenfell *et al.* [3], which relates the risk of immune evasion at the within-host level to the strength of immunity. While the concept has proved influential [2,4–7 ], there have been few quantitative realizations to date. A notable within-host exception is given in [8] and, importantly Ferguson *et al*. and Koelle *et al*. [9,10] address strain-replacement in influenza, focusing on the impact of strain-transcending immunity and complex fitness landscapes, respectively. In [11], the authors explored a model of the dynamics of antigenic distance (and thus immune escape) within a single influenza season. Perhaps the clearest demonstration of the phylodynamic curve is due to Chabas *et al*. [12], who performed experiments with bacteriophages and varying population fractions of CRISPR-resistant host bacteria showing that the probability of observing escape mutations was maximized for intermediate levels of host resistance. They also modelled the phenomenon, focusing mainly on how the fraction of resistant hosts, as well as the heterogeneity in resistance alleles, affected the risk of escape mutations emerging in a static population. We argue that there is a further need for generic modelling frameworks to explore the relationship between individual and population-level epi-evolutionary dynamics. In particular, there is a need for models of emergence risk which allow for time-varying ecological dynamics, including exogenous factors affecting transmission, such as interventions and seasonality. Aiming for a broadly applicable, reductionist framework addressing this gap, we develop a mathematical description of the *net viral adaptation rate* (the immune escape variant emergence risk per unit of time) as shaped by population immunity and transmission (see figure 1B).

The phylodynamic curve was originally formulated as a statement on within-host adaptation, encapsulating the nonlinear relation between the strength of immunity and the risk of a viral escape variant arising during infection. At low levels of immune pressure, viral populations may be large (high abundance) but they face minimal selective pressure and so adaptation is limited. Conversely, as the immune pressure intensifies, the selection for immune escape variants becomes more pronounced, accelerating the rate of viral adaptation. However, beyond a certain threshold, robust immune responses reduce the viral population size enough to restrict the pool of genetic variants and slow adaptation. Consequently, the net adaptation is expected to peak at intermediate levels of strength of immunity. Together, these dynamics produce an inverted U-shaped phylodynamic curve, when the net adaptation rate is plotted against the immune strength.

In this study, we take a population-level view of the phylodynamic curve and consider how the risk of immune escape depends on factors such as population immunity (including factors such as leaky immunity and partial cross-immunity), infection prevalence, transmissibility, rates of case importation, etc. The inclusion of imperfect (‘leaky’) immunity, a phenomenon that was recently demonstrated in COVID-19 by Lind *et al*. [13], turns out to be especially important, as it has profound consequences for the risk of immune escape variant emergence. We provide a quantitative, population-level realization of the phylodynamic curve in stationary situations (such as endemic equilibrium) as well as a framework for the computation of the population-level net adaptation rate in arbitrary dynamic settings such as seasonally driven epidemics, during variant replacement or co-circulation; see figure 1 for a schematic. The general and extensible framework introduced here allows us to probe the complex dynamics of pathogen–host interactions insofar as they affect the risk of immune evasion.

The findings presented here, and the approach more generally, hold implications for public health, including vaccine design and distribution as well as infectious disease control. Understanding the conditions which lead to a heightened risk of escape variant emergence—i.e. the peak of the phylodynamic curve—is crucial. By pinpointing the conditions under which pathogens are most likely to evolve to escape immunity helps prioritize interventions, whether they be in the form of vaccination campaigns, public health policies, non-pharmaceutical interventions or therapeutic approaches.

The phylodynamic curve highlights the heightened potential for variant emergence at intermediate strength of immunity. In accordance with that, the evolutionary consequences of weakly protective immune responses have recently been highlighted by the evolution of SARS-CoV-2, where novel variants tended to represent large genomic jumps relative to the resident variant [14], and a growing body of evidence points to insufficient antibody responses as a key factor in the emergence of these divergent escape variants [15–20].

After introducing the central mathematical model in §2, we go on to discuss the net adaptation rate in equilibrium in §3. We begin by considering the simplest scenario, namely the limit of highly durable (albeit imperfectly infection-blocking) immunity. We then consider the more general endemic equilibrium scenario, where immunity duration is finite and protection is imperfect. We then conceptually apply the proposed framework to several different pathogens, characterized by different degrees of transmissibility, strength of infection and antigenic constraints. A quantitative application of the framework across multiple pathogens may open the door to comparative phylodynamics, improving our understanding of why some pathogens readily evolve immune-evading strains, while others do not.

After these equilibrium considerations, we analyse out-of-equilibrium dynamics of the net adaptation rate in §4. We consider seasonally driven transmission and show how importation of cases from less seasonally varying regions can qualitatively shift the timing of variant emergence. Finally, motivated by the COVID-19 pandemic, we discuss some likely impacts of transmission-reducing non-pharmaceutical interventions (NPIs) on the risk of immune escape variant emergence while the NPIs are in force, as well as after they are lifted.

## 2. Methods

In this section, we derive analytical expressions for the net adaptation rate, defined as the probability per unit time that a viral immune escape variant will emerge. The expression for the net adaptation rate depends on the precise underlying epidemiological model and assumptions made, but the derivations follow similar steps in each case.

First, we derive the net adaptation rate in the situation where there is a single resident strain and examine special cases of this scenario.

Second, we present the net adaptation rate in the presence of two strains with partial cross-immunity, as well as a system of ordinary differential equations describing the dynamics of the strains. In this case, the net adaptation rate describes the probability rate for a *third* (immune-evading) strain emerging.

### 2.1. The net adaptation rate

Assume that a *resident strain* is circulating, with known densities of susceptible (S(t)), infected (I(t)) and recovered individuals (R(t)). Note that these may be time-dependent functions, and are subject to S(t)+I(t)+R(t)=1. We take immunity to be imperfect (*leaky*), meaning that recovered individuals are still partially susceptible, their susceptibility being discounted by a factor of 1−σ. We denote σ the *strength of immunity*.

Each infected individual causes new infections at a rate ((1−σ)R+S)β. Immune-evasion mutations are assumed to arise at transmission, with a per-transmission rate of μ. Such mutations thus appear in the population at a rate ((1−σ)R+S)μβI. The net adaptation rate measures the rate at which immune-evading mutations appear *and* establish in the population. Thus, we must compute the probability that a newly arisen mutant variant survives (avoids stochastic extinction) or, conversely, the *extinction probability*. To do this, we can use the equation for the extinction probability d of a Galton–Watson branching process [21,22]:

(2.1) $$\begin{array}{lrr} & d=\sum_{i=0}^{\infty }P_{i}d^{i}, & \end{array}$$

where $P_{i}$ is the offspring distribution of the mutant variant, and the right-hand side may be recognized as the probability generating function G(d) associated with $P_{i}$. The equation for the extinction probability can thus be succinctly written as d=G(d), which is, in general, a transcendental equation.

Since infectious *time* is assumed to follow an exponential distribution in typical compartmental models and the infection process itself is Poissonian, the offspring number will follow a k=1 negative binomial distribution (also sometimes referred to as a geometric distribution) with mean (reproductive number) $R_{\text{eff}}^{\text{mut}}=(S+(1-\sigma_{c})R)\beta /\gamma$, with γ the recovery rate and $\sigma_{c}$ the strength of cross-immunity between the potential escape variant and the resident strain. In this case, the extinction probability is given by [23]


(2.2) $$\begin{array}{lrr} & d=1/R_{\text{eff}}^{\text{mut}}. & \end{array}$$

This relation holds for $R_{\text{eff}}^{\text{mut}}\geq 1$. Consequently, one could write $d=1/\text{max}(1,R_{\text{eff}}^{\text{mut}})$, but we will keep the simpler expression and simply keep in mind that it describes variants with reproductive numbers exceeding 1, since extinction is guaranteed for $R_{\text{eff}}^{\text{mut}}<1$. In the case discussed above, the extinction risk is thus simply


(2.3) $$\begin{array}{lrr} & d=\frac{\gamma }{(S+(1-\sigma_{c})R)\beta }. & \end{array}$$

Combining these elements, the net adaptation rate $r_{a}$ is given by


(2.4) $$r_{a}=((1-\sigma )R+S)\;\mu \beta I\;(1-d).$$

Formally, the extinction risk d given above assumes a homogeneous branching process in which the offspring distribution does not change with time. We discuss this simplification in electronic supplementary material, section A1, and probe its accuracy in electronic supplementary material, section A2.

Incorporating an influx of mutations from outside the modelled population amounts to adding a density-independent term to the factor ((1−σ)R+S)μβI that describes the rate at which escape mutations are generated, rendering it $((1-\sigma )R+S)\;\mu \beta I+\mu_{im}$. The final net adaptation rate then becomes


(2.5) $$r_{a}=\left[\left((1-\sigma )R+S\right)\;\mu \beta I+\mu_{im}\right]\;(1-d).$$

Note that, while $\mu_{im}$ is presently assumed independent of the local densities S, I and R, it may be modulated by,for example, the incidence at an adjacent location from which the imported cases originate.

The dynamical variables S, I and R appear in (2.5) and must be supplied before the net adaptation rate can be computed. Importantly, these fractions may be supplied by empirical time series *or* by a dynamical model. In the present work, we focus on model-supplied time series but comment on the prospects of applying the framework to empirical time series in §6.

Since the population fractions will be model-derived, we require an epidemiological model with imperfect (leaky) immunity, in line with the assumptions made in the derivation of (2.5). We will employ a compartmental ODE model including waning, leaky immunity, which we denote SIR(S/I), i.e. a model in which the trajectory upon infection is to transition from susceptible to infected and then to recovered, from where it is possible to become susceptible again (due to waning immunity) or re-infected due to the imperfect strength of immunity (parameter σ). The equations of the SIR(S/I) model are


(2.6)dSdt=−βSI+δR,(2.7)dIdt=βSI+(1−σ)βRI−γI

and

(2.8) $$\begin{array}{lrlr} & \frac{\text{d}R}{\text{d}t} & =\gamma I-\delta R-(1-\sigma )\beta RI. & \end{array}$$

It should be noted that, in our simulations, we will also be interested in scenarios where the transmission rate is seasonally driven. Thus, in addition to the variables S(t), I(t) and R(t), the transmission rate β=β(t) may depend on time as well. See table 1 for an overview of the parameters of the SIR(S/I) model as well as those which appear in the net adaptation rate. A state diagram of this model can be found in electronic supplementary material, figure S5A.

**Table 1.** Parameters used in the SIR(S/I) model and associated net adaptation rate equation.

parameter	description	allowed values
β	transmission rate	[0;∞)
γ	recovery rate	[0;∞)
σ	strength of immunity	[0;1] (fractional reduction in susceptibility following infection)
δ	immunity waning rate	[0;∞) (0 is durable immunity, ∞ is instantaneous waning)
$\sigma_{c}$	strength of cross-immunity	[0;1] (fractional reduction in susceptibility to escape variant following infection with resident variant)

As is clear from the above, the SIR(S/I) model yields only the dynamics of the resident strain. This is because we will, in the majority of this article, be concerned only with the *risk of emergence* of an immune-evading strain on the background of a resident strain. However, in §2.2, we do extend the framework to evaluate cases where a new strain *does* emerge and circulates at appreciable levels.

#### 2.1.1. Special case: endemic equilibrium

The endemic equilibrium value of the infected fraction ($I^{*}$) in the SIR(S/I) model can be determined from the equation


(2.9) $$\begin{array}{c} f(I^{*})\;=\;A_{2}(I^{*}{)}^{2}\;+\;A_{1}I^{*}\;+\;A_{0}=0,\\ A_{2}\;=\;-\beta (1-\sigma ),\;\;A_{1}=-\delta +\beta (1-\sigma )-\gamma ,\;\;A_{0}=\delta (1-\gamma /\beta ). \end{array}$$

The endemic equilibrium values of the susceptible ($S^{*}$) and recovered ($R^{*}$) fractions can then be determined from


(2.10) $$\begin{array}{lrlr} & S^{*} & =\frac{\delta (1-I^{*})}{\beta I^{*}+\delta }, & \end{array}$$

(2.11) $$\begin{array}{lrlr} & R^{*} & =1-(S^{*}+I^{*}). & \end{array}$$

Given the steady-state values $S^{*}$, $I^{*}$ and $R^{*}$, the equilibrium net adaptation rate can thus be computed.

#### 2.1.2. Special case: complete exposure

A particularly simple special case occurs when the entire population has (at some point) been exposed to the resident strain. This may approximately apply for a disease that produces long-lasting albeit imperfectly infection-blocking immunity. In this case, all individuals are either in compartment R or I, and the net adaptation rate in the absence of imported cases is given by $r_{a}=\left(1-\sigma )(1-R)R\mu \beta (1-d\right)$, where we used R+I=1. The extinction probability d is then derived from a k=1 negative binomial offspring distribution with mean $R_{\text{eff}}^{\text{mut}}=(1-\sigma_{c})R\beta /\gamma$.

Since the system is assumed to be at equilibrium, we can solve for the steady-state values of the recovered and infection fractions, $R^{*}$ and $I^{*}$, using


$$\begin{array}{rl} \frac{(1-\sigma )\beta R^{*}}{\gamma } & =1,\\ I^{*}+R^{*} & =1, \end{array}$$

which lead to


$$\begin{array}{rl} R^{*} & =\frac{\gamma }{(1-\sigma )\beta },\\ I^{*} & =1-\frac{\gamma }{(1-\sigma )\beta }. \end{array}$$

Inserting this in the net adaptation rate yields


(2.12) $$r_{a}=\mu \gamma \;\left(1-\frac{\gamma }{(1-\sigma )\beta }\right)\;(1-d)=\mu \gamma \;\left(1-\frac{\gamma }{(1-\sigma )\beta }\right)\;\frac{\sigma -\sigma_{c}}{1-\sigma_{c}},$$

where we used that, in this model


(2.13) $$\begin{array}{lrlr} & d & =\frac{\gamma }{(1-\sigma_{c})R^{*}\beta }=\frac{1-\sigma }{1-\sigma_{c}}, & \end{array}$$

which follows from equation (2.5). Note that the conditions $I^{*}\geq 0$ (which implies σ≤1−γ/β) and d≤1 (which implies $\sigma_{c}<\sigma$) together guarantee the positivity of $r_{a}$.

### 2.2. Strain co-circulation

This section tackles the situation where two strains (‘1’ and ‘2’) are circulating at appreciable levels. The resulting model can be used to model the epidemiological dynamics once an immune-evading strain *does* emerge, and to evaluate the risk of another immune-evading variant emerging once two variants circulate. In the following, we use $R_{i}$ (for i∈{1,2}) to refer to individuals recovered from infection with strain i only, while the compartment R contains individuals who have previously been infected by both types. The infected compartments are of the form $I_{i}$, corresponding to primary infection with strain i, and $I_{ij}$ (with i≠j), corresponding to infection with strain i, having previously been infected with strain j (and potentially with i itself).

Each infection with strain 1 causes new infections at a rate


$$\begin{array}{r} (S+(1-\sigma )\;(R_{1}+R)+(1-\sigma_{c})\;R_{2})\beta , \end{array}$$

and similarly for strain 2, except with indices 1 and 2 swapped. Mutations due to strain 1 thus arise at a rate


$$\begin{array}{r} \left(S+(1-\sigma )\;(R_{1}+R)+(1-\sigma_{c})\;R_{2})\;\mu \beta \;(I_{1}+I_{12}\right) \end{array}$$

and similarly for strain 2. The net adaptation rate (with a rate of importation, $\mu_{im}$) in the presence of two strains is thus given by

(2.14) $$\begin{array}{ll} r_{a} & =[\left(S+(1-\sigma )(R_{1}+R)+(1-\sigma_{c})\;R_{2})\;\mu \beta \;(I_{1}+I_{12}\right)\\ & \;\;\;+(S+(1-\sigma )(R_{2}+R)+(1-\sigma_{c})\;R_{1})\;\mu \beta \;(I_{2}+I_{21})+\mu_{im}](1-d) \end{array},$$

where $d=1/R_{\text{eff}}^{\text{mut}}$ is given by

(2.15) $$\begin{array}{rl} d & =\frac{\gamma /\beta }{(1-\sigma_{c})(R_{1}+R_{2}+R)+S} \end{array}.$$

Note that (2.15) assumes that the potential-emerging strain exhibits the same strength of cross-immunity $\sigma_{c}$ towards the two circulating strains as these do to each other. This assumption, however, can be easily modified by replacing $\sigma_{c}$ in (2.15) by a distinct cross-immunity strength.

Until strain 2 emerges, the net adaptation rate is thus described by (2.5), and subsequently by (2.14) (which then describes the per-time risk of a third strain emerging). The system of ordinary differential equations for the two-strain model to which we couple (2.14) is


(2.16)dSdt=−βS(I1+I12+I2+I21)+δ(R1+R2+R),(2.17)dI1dt=βS(I1+I12)+β(1−σ)R1(I1+I12)−γI1,(2.18)dI2dt=βS(I2+I21)+β(1−σ)R2(I2+I21)−γI2,(2.19)dR1dt=γI1−δR1−β(1−σ)R1(I1+I12)−βR1(I2+I21),(2.20)dR2dt=γI2−δR2−β(1−σ)R2(I2+I21)−βR2(I1+I12),(2.21)dI12dt=β(1−σ)R(I1+I12)+βR2(I1+I12)−γI12,(2.22)dI21dt=β(1−σ)R(I2+I21)+βR1(I2+I21)−γI21

and

(2.23) $$\begin{array}{ll} \frac{\text{d}R}{\text{d}t} & =\gamma (I_{12}+I_{21})-\delta R-\beta (1-\sigma )R\left(I_{1}+I_{12}+I_{2}+I_{21}\right) \end{array}.$$

A state diagram of this model can be found in electronic supplementary material, figure S5B.

## 3. Equilibrium adaptation rate

In this section, we explore how the net adaptation rate, i.e. the risk of immune escape, depends on the strength of immunity and transmissibility in steady-state scenarios.

### 3.1. Complete exposure

**Figure 2.**
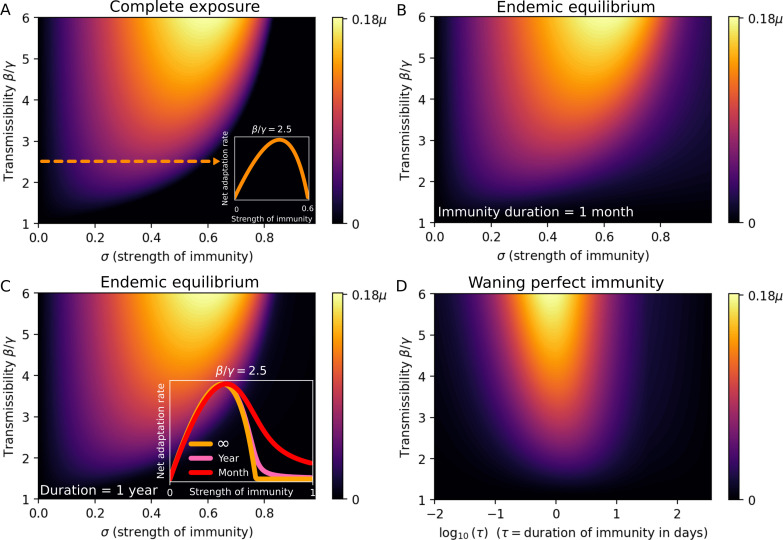
The net adaptation rate in equilibrium scenarios. (A) The complete exposure scenario, with net adaptation rate given by (2.12). For each value of the transmission rate β, a non-monotonic dependence on the strength of immunity σ results. The inset shows the phylodynamic curve that results at β=2.5γ corresponding to an $R_{0}$ of 2.5 at σ=1. (B) Endemic equilibrium at a high rate of immunity waning (δ=1/month). (C) Waning perfect immunity in endemic equilibrium. The net adaptation rate $r_{a}$ as a function of the transmission rate β and duration of immunity, τ=1/δ. At fixed β, a non-monotonic dependence on δ appears, but maximal $r_{a}$ occurs at very short immunity duration (approx. 1 day), meaning that the net adaptation rate is a monotonically increasing function of the waning rate δ at all epidemiologically relevant values of δ, when perfectly infection-blocking immunity (σ=1) is assumed. (D) Endemic equilibrium at a moderate rate of immunity waning ($\delta =1\;{\text{yr}}^{-1}$). Note that this longer lasting scenario (relative to panel C) is quite similar to the complete exposure scenario of panel (A). The recovery rate is fixed at γ = 0.5 d^−1^ throughout this figure.

We explore two separate equilibrium scenarios of increasing complexity. The first is that of a population that has been completely exposed to the resident variant, meaning that all individuals are either recovered or currently infected. This scenario approximates either the situation after a highly transmissible variant has swept the population, or a disease with long-lasting (albeit imperfect) immunity, to which most individuals beyond a certain age would have been exposed. Just as importantly, this scenario also covers the situation where the population has been vaccinated with a vaccine that does not provide perfect protection against infection [24,25]. Due to its simplicity, this scenario allows for an especially straightforward interpretation. The resulting family of phylodynamic curves (one for each value of the transmission rate β) is shown in figure 2A. The inset shows the inverse U-shaped phylodynamic curve that emerges as different values of the strength of immunity σ are scanned at a fixed transmission rate β.

Equation (2.12) reveals that, at weak immunity (low σ), the net adaptation rate is limited by the mutant survival chance (which simply equals σ when cross-immunity is negligible), while at strong immunity (σ close to the herd immunity threshold $1-1/R_{0}$), the infected fraction is the limiting factor.

In the electronic supplementary material, we numerically simulate the complete exposure scenario with explicit stochasticity (i.e. without making any analytical assumptions about the extinction risk, d) via the Doob–Gillespie algorithm [26]. In this case, we are able to explicitly measure the net rate of adaptation as the reciprocal of the average time before an immune escape variant emerges. The results can be found in electronic supplementary material, figure S3, and show excellent overall agreement with figure 2A.

### 3.2. Endemic equilibrium

We now turn to the endemic equilibrium solution of the full SIR(S/I) model, which includes waning *im*perfect immunity (i.e. σ<1). Note that the complete exposure scenario previously studied is formally the long-term (equilibrium) solution to the SIR(S/I) model in the limit where δ tends to 0. Figure 2B,C shows the net adaptation rate (as a function of β and σ) at two different rates of waning, corresponding to mean durations 1/δ=1month and 1/δ=1 year, respectively. The recovery time is 1/γ=5days, and we note that already at an immunity duration of one month, the plot bears a strong resemblance to the complete exposure result of figure 2A, and the two are almost indistinguishable at an immunity duration of 1 year. The inset of figure 2C gives the net adaptation rate profiles at fixed transmissibility. The profiles show that some qualitative differences do arise when waning is fast—the curve corresponding to a one month duration remains non-zero even at σ=1, consistent with the corresponding point in figure 2C (around β/γ=2.5 and $\log_{10}(\tau )=1.48$).

### 3.3. Evolutionary consequences of waning immunity versus leaky immunity

In the previous sections, we have assumed that immunity is imperfect, in the sense that it reduces susceptibility by a factor (1−σ) rather than completely. This is in contrast to most theoretical models of how the evolution of immune escape is shaped by population immunity, which have typically assumed that homologous immunity is perfect while it lasts [27–29], and that variable ‘strength’ of immunity can be captured by varying the *duration* of protection. In this section, we will adopt the assumption that homologous immunity is perfectly infection-blocking while it lasts, and is thus limited only by exponential waning. In this case, immunity is then binary: either one is perfectly protected against infection, or one is fully susceptible [30]. We explore this assumption to see how it affects conclusions regarding the net adaptation rate, and thus the evolution of immune escape.

Assuming endemic equilibrium, we explore the effects of waning immunity in figure 2D—note the logarithmic horizontal axis. While a non-monotonic relation between immunity duration and the net adaptation rate does appear, the maximum occurs at an immunity duration on the order of a single day, a waning so rapid as to be epidemiologically irrelevant. We thus conclude that exponentially waning perfect immunity does not yield an inverse U-shaped phylodynamic curve, and that within epidemiologically relevant parameters, faster waning tends to lead to faster adaptation. This finding may lend itself to at least crude empirical testing, by comparing pathogens with different rates of immune waning. However, it should be mentioned that this result is likely to depend, to some degree, on assumptions made about the per-generation rate of antigenic evolution versus the per-infected-time rate—an aspect that warrants exploration in future works. Overall, we are led to conclude that the distinction between leaky immunity and waning but polarized (all-or-nothing [30]) immunity has profound consequences for the emergence of escape variants, highlighting the need for mapping the individual-level post-exposure (and post-vaccination) protection against reinfection for circulating pathogens. While leaky immunity has been demonstrated in COVID-19 [13], a challenge study with infectious haematopoietic necrosis virus in rainbow trout found very heterogeneous vaccinal immunity, in an almost all-or-nothing fashion [31], emphasizing the multiple ways in which protection may be imperfect.

### 3.4. The role of antigenic constraint and the effective mutation rate

Due to the simplicity of the proposed framework, molecular aspects of antigenic evolution are not explicitly modelled and as a consequence, the within-host evolutionary process is highly coarse-grained. However, through the effective mutation rate μ, which parametrizes the per-transmission risk of an immune-evading mutation occurring, we can probe some of the overall effects of antigenic constraint.

Despite facing strong selective pressure and maintaining substantial viral abundance, some viruses exhibit limited antigenic variability [32,33]. This situation is well exemplified by measles. With its high basic reproductive number, strong, durable immunity and relatively widespread vaccination, measles would be expected to present ideal conditions for the emergence of escape variants. However, such variants are not observed. This phenomenon can largely be attributed to ‘antigenic constraint’, where biophysical limitations restrict the virus’s capacity for viable mutations. In the case of measles, these constraints might arise from conformational limitations of its primary antigens, where substantial alterations could impede the virus’s infectivity or replication efficiency. Despite observed genetic variability in the F (*fusion*) and H (*hemagglutinin*) envelope proteins of measles, analysis has shown that existing constraints only allow for a single serotype [34]. The consequences of these constraints are remarkable, exemplified by the fact that a genotype capable of evading vaccine-induced immunity has not emerged after decades of widespread vaccination [35]. Serum neutralization assays with mutated H proteins have shown that several co-dominant glycoprotein epitopes exist within this protein and that the H protein is co-dominant with respect to serum neutralization with the F protein, meaning that a large number of mutations (each with potential fitness costs) would be required to effectively evade immunity [35,36].

Similarly, influenza A/H3N2’s antigenic evolution is not without its bounds. The evolution of its hemagglutinin has been shown to be shaped by geometric constraints [37]. And as detailed in [38], constraints on net charge—maintained by the balancing of charged amino acids—limit the evolution of the neuraminidase antigenic region studied. A similar result has been found in the hemagglutinin of influenza A/H1N1 [39].

**Figure 3.**
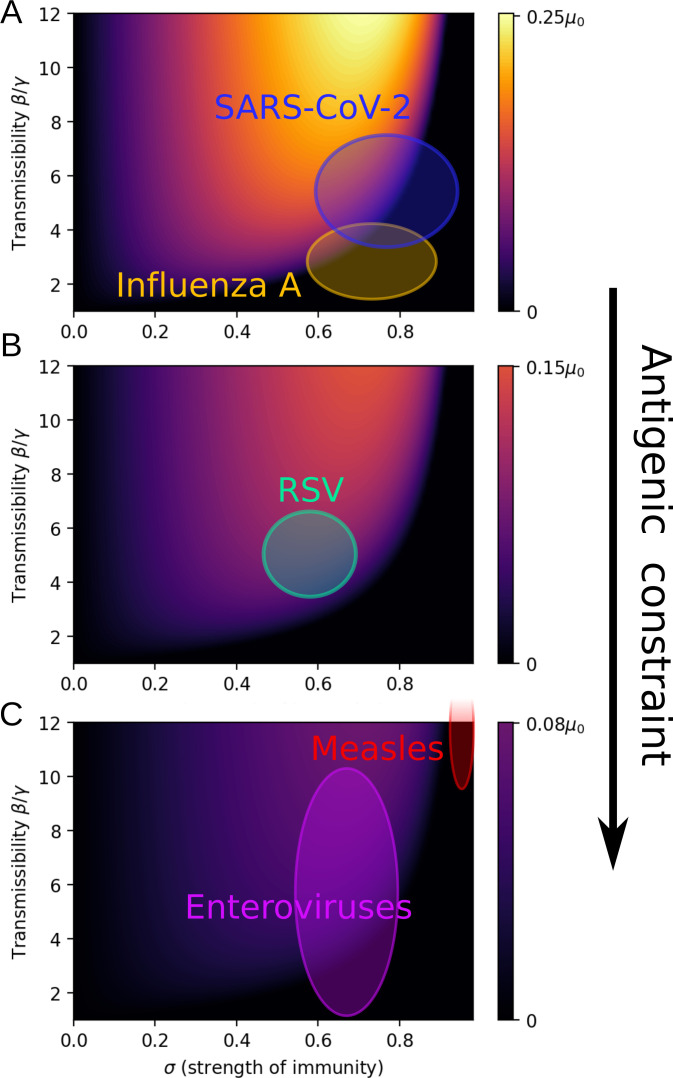
A conceptual phylodynamic landscape, parametrized by transmissibility β, strength of immunity σ and effective mutation rate μ. Stronger antigenic constraints decrease the effective mutation rate, as indicated by the vertical arrow. The colour indicates the (population-level) net adaptation rate. (A) μ=1, (B) μ=0.6, (C) μ=0.3. The adaptation rate is computed based on the complete exposure equilibrium scenario and the recovery rate is fixed at γ = 0.5 d^−1^ throughout this figure. In this figure, no cross-immunity between the resident variant and a putative escape variant is assumed. Electronic supplementary material, figure S2, explores the impacts of partial *cross*-immunity.

Together, these examples illustrate an important aspect of pathogen evolution: the balance between selective pressures and the inherent structural and functional constraints of the pathogen. In our model, we only very crudely incorporate antigenic constraints, and do so via an effective description: we subsume their effects into the μ parameter, the antigenic mutation rate. We explore the effects of this parameter in figure 3, where we have conceptually placed a number of pathogens in a multidimensional net adaptation rate landscape, parametrized by the effective mutation rate μ, the transmission rate β and the strength of immunity σ. We stress that this rough overview is only meant to show how the proposed framework can be used to create an easily interpretable immune escape risk classification. The figure assumes equilibrium (under the complete exposure scenario previously presented in figure 2A) and thus does not reflect, for example, the risk of novel variants upon introduction to a naive population, nor the appearance of variants driven by higher intrinsic transmissibility (rather than immune escape).

In table 2, we list the parameters used in creating figure 3. For the strength of the immunity parameter σ, we have prioritized challenge studies, since population-level and registry studies often have difficulty distinguishing between leaky and merely short-lived immunity. The basic reproduction number for influenza (R_0 _≈ 1.4–2.8) was based on [40]. $R_{0}$ of course refers to the reproductive number in a naive population. For influenza, this is best approximated by measurements of pandemic influenza, while seasonal influenza typically fails to realize this figure, precisely because of partial immunity. For enteroviruses, a very diverse class, estimates of $R_{0}$ vary widely. Park *et al.* [52 ] obtain estimates for EV-D68 in the 10−20 range, while Ma *et al*. [50 ] estimate the values 5.5 for EV-A71 and 2.5 for Cox A16. Lim *et al*. [51 ] report an $R_{0}$ of 5.0 for Cox A6, 2.4 for Cox A16, 2.4 and 3.5 for EV-A71. For respiratory syncytial virus (RSV), estimates are also highly variable. Reis & Shaman [45] find a mean literature estimate of approximately 4.5 while they themselves estimate it to 2.8. Reis & Shaman [46] estimate $R_{0}$ at 3.0, while Pitzer *et al.* [47 ] find a higher value of 8.9. SARS-CoV-2 $R_{0}$ values vary by variant, with the ancestral strain estimated at around 3 [42 ], while the $R_{0}$ of the Omicron variant has been estimated at 8−9 [43 ].

**Table 2.** Parameters used in figure 3 and their origins.

pathogen	basic reproductive number	strength of immunity	antigenic constraint
influenza A	1.4−2.8 [40]	≈60% [41] (to identical strain)	assumed low
SARS-CoV‐2	ancestral strain: ≈3 [42]. Omicron: 8−9 [43]	≈80% [44]	assumed low
RSV	3−5 [45,46] ([47] finds 8.9)	50−75% [48,49]	assumed moderate
enteroviruses	2.5−5.5 [50,51] (up to 20 for EV-D68 [52])	high, although reinfections with same serotype occur [53]	assumed high
measles	10−18 [54]	high and life-long [55,56]	high [34–36]

## 4. Non-equilibrium dynamics

In this section, we allow full temporal variation in the susceptible, infected and recovered population fractions, and allow for time-dependent transmission rate β(t).

### 4.1. Seasonality, imported cases and variant emergence

**Figure 4.**
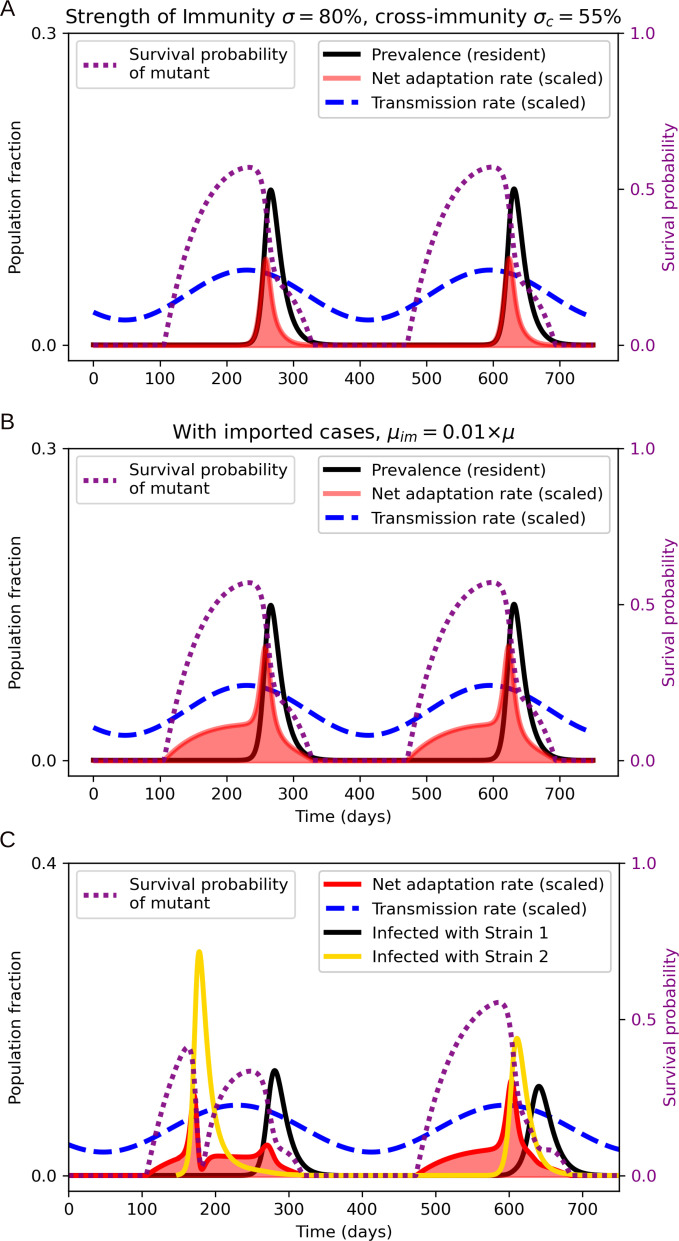
Seasonality and the emergence of an escape variant. Sinusoidally modulated transmission rate (dashed blue curve). (A) No imported cases ($\mu_{\text{im}}=0$). The bulk of the emergence risk (area under the red curve) occurs during the epidemic growth phase, while incidence is high and the susceptible fraction is substantial. (B) Constant importation rate $\mu_{\text{im}}=0.01\mu$, modelling imports from a region with year-round circulation. The trough period now sees a substantial emergence risk. (C) Full two-strain model. An immune-escape variant (yellow prevalence curve) emerges at time t=150 days and disrupts the seasonal pattern. The moderate cross-protection ($\sigma_{c}=0.55$) leads to subsequent co-circulation.

Seasonal variation in transmission rate not only leads to periodic outbreaks but to a strongly time-varying net adaptation rate as well. Figure 4 shows the net adaptation rate as given by (2.5) coupled to the system of differential equations (2.3)–(2.5) describing the SIR(S/I) model, with sinusoidally varying transmission rate $\beta (t)=\beta_{0}(1+0.2\cos(2\pi t\;{\text{yr}}^{-1}))$. In figure 4A, the importation rate is turned off ($\mu_{\text{im}}=0$), a scenario which leads to the bulk of the emergence risk occurring during the growth phase of the seasonal epidemic (note that the risk of emergence is very low during the decreasing phase of the epidemic, echoing a result by [57]). This is in contrast to the case in figure 4B, where a constant importation rate is turned on ($\mu_{\text{im}}=0.025\mu$) and a large part of the emergence risk (understood as the area under the red curve) occurs during the *trough period*, i.e. while prevalence is locally practically non-existent. This shows one of the potentially profound consequences of year-round reservoirs for otherwise seasonal pathogens. A salient example may be influenza A, which is strongly seasonal in temperate climates, while tropical regions exhibit extensive year-round influenza activity as well as irregular timing of outbreaks [58–61].

In figure 4C, the two-strain model described by equations (2.16)–(2.23) is implemented, and an immune escape variant ( ‘strain 2’) emerges at time t=150 days. The surge of the new strain disrupts the seasonal pattern and the moderate level of (symmetric) cross-protection ($\sigma_{c}=0.55$) allows for subsequent co-circulation of the newly emerged and the previously dominant strain ( ‘strain 1’). It is not just the pattern of circulation that is disrupted—so is the net adaptation rate, exhibiting two peaks within a single season. The simulation of figure 4C also highlights a central aspect of the proposed model framework: the net adaptation rate describes the *potential* for adaptive sweeps, but once such a sweep occurs, it will strongly perturb the net adaptation rate (as well as affect the epidemiological dynamics).

Due to seasonality, the timing of variant emergence has a large influence on the subsequent dynamics. We note that, if a variant emerges during the first half of the trough following a seasonal outbreak, it can cause a small outbreak of the novel variant, followed by a large outbreak once growth conditions improve (i.e. once the transmission rate increases again). This could be a potential mechanism of herald waves in, for example, pandemic influenza [62–64] and other pathogens [65]. A similar notion is explored in the context of cholera in [65].

### 4.2. The impact of non-pharmaceutical interventions

The NPIs, such as lockdowns, implemented during the COVID-19 pandemic were often highly effective in reducing the COVID-19 transmission rate [66], as well as those of other circulating respiratory pathogens [67–73]. While the short-term epidemiological implications for COVID-19 are reasonably well understood, the effects on pathogen evolution are likely complex and remain relatively poorly understood, especially as it pertains to pathogens other than COVID-19, whose dynamics was also affected by interventions.

In this section, we first explore how NPIs acutely affect the net adaptation rate in a few simple situations.

**Figure 5.**
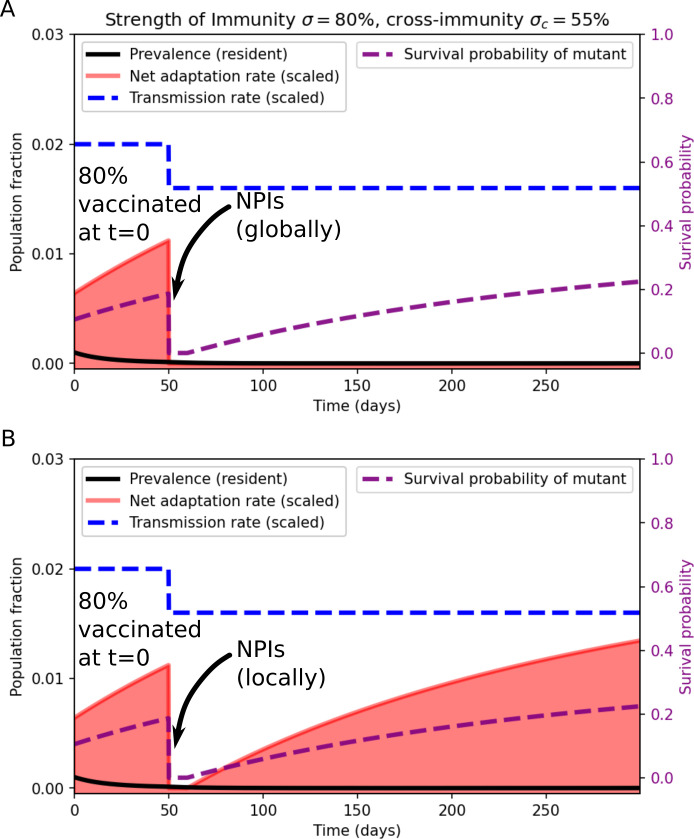
Impact of non-pharmaceutical interventions on the net adaptation rate (red curve), when applied globally (A) and locally (B). In the local case, a constant importation rate of $\mu_{im}=0.01\mu$ is assumed.

In figure 5, we assume that 90% of the population are initially vaccinated with a moderately effective vaccine that reduces susceptibility by 80% against the resident strain and by 55% against a potentially emergent strain. We also assume that immunity (vaccine-induced as well as infection-derived) wanes at a rate of $1\;{\text{yr}}^{-1}$. We include the initial vaccination to show the effects of immune waning during periods where no (or very little) circulation is taking place locally. At t=50 days, NPIs are introduced either globally (figure 5A) or only locally (figure 5B), lowering the transmission rate β (blue dashed curve in Figure 5). In the former case, NPIs are able to keep the net adaptation rate at essentially zero, since the implemented interventions effectively limit the viral abundance. In the latter case, NPIs initially hamper adaptation, but once immunity wanes even moderately, the net adaptation rate begins to increase. This is due to the favourable growth conditions created for an invading escape variant—no major competition from a resident variant, but sufficient numbers of fully or partially susceptible individuals for a somewhat immune-evading variant to invade. The importation rate from neighbouring (intervention-free) regions then continuously provides the ‘sparks’ that can ignite such an invasion. In this sense, imported cases effectively bypass the low local viral abundance that would otherwise limit the net adaptation rate [74].

**Figure 6.**
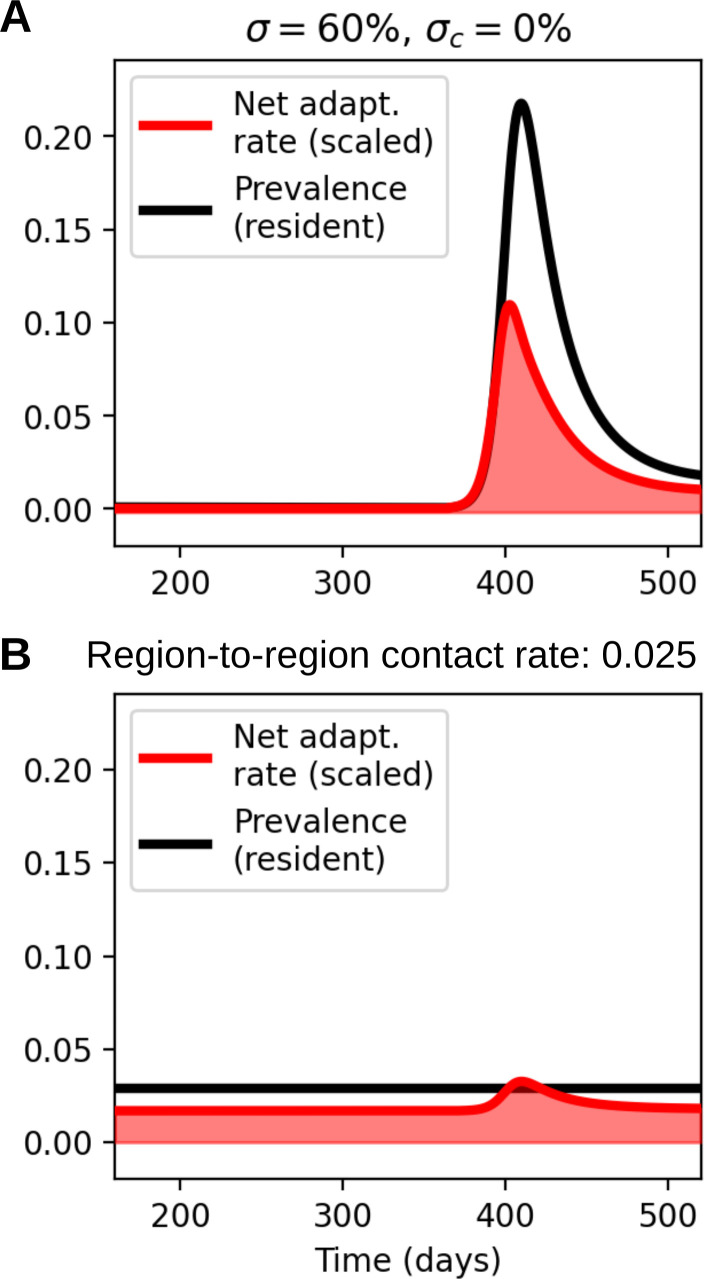
Net adaptation rate dynamics (of non-COVID respiratory pathogen) once NPIs are lifted, in the region previously under NPIs (the *post-NPI* region) (A) and in an adjacent region not previously subjected to extensive restrictions (the *never-NPI* region) (B). The importation rate in the never-NPI region is assumed proportional to the prevalence in the post-NPI region: $\mu_{\text{im}}(t)=r_{c}\mu I_{\text{post-NPI}}(t)$, with $r_{c}=0.025$ a contact rate from the post-NPI to the never-NPI region.

Having considered the acute effects of non-pharmaceutical interventions, i.e. the effects they have while they are in force, we move on to the question of what happens once NPIs are lifted and the transmission rate increases again. We will specifically consider the effects on non-COVID-19 pathogens, which were also affected by NPIs but were not as extensively vaccinated against during the NPI period. The lifting of restrictions has led to surges in several pathogens [75] due to the build-up of susceptibles during the NPI period. We find (figure 6A) that such a surge is accompanied by a rapidly rising net adaptation rate, despite the initially low immune pressure.

However, viewing a previously locked-down region in isolation hides the effects that such a local surge may have on neighbouring regions with different levels of population immunity. Specifically, regions that did not experience extensive lockdowns may have appreciable population immunity towards the pathogen in question, and correspondingly different selection pressures. To model this, we consider a region that never implemented NPIs (the *never-NPI region*), and in which the disease in question is instead in an endemic equilibrium. During the surge in the previously locked-down region (the *post-NPI region*), some spill-over to neighbouring regions is likely. Thus, we model an importation rate in the never-NPI region that is proportional to the prevalence in the post-NPI region: $\mu_{\text{im}}(t)=r_{c}\mu I_{\text{Post-NPI}}(t)$, with $r_{c}$ a contact rate from the post-NPI to the never-NPI region. We observe that, although the prevalence in the never-NPI region stays constant, the region-to-region contact leads to an appreciably elevated net adaptation rate in the never-NPI region as well (figure 6B).

The impact of cross-immunity (infection-derived as well as vaccine-induced) that reduces susceptibility (and infection duration) on the growth rate of an invading variant and the eventual depth of the trough it undergoes was considered by Restif & Grenfell [76]. However, the authors do not consider imperfect *homologous* immunity, which we have found to be an important factor in shaping the risk of immune escape. It is worth noting that they find that the depth of the trough that an invading variant undergoes follows a U-shaped curve as a function of strength of immunity (as opposed to the *inverse* U-shaped phylodynamic curve). This is expected: highly successful variants tend to overexploit the ‘resources’ (susceptibles), and then undergo a severe trough. However, in practice, deep local troughs are typically not enough to eradicate a novel escape variant, since out-of-phase outbreaks will typically occur elsewhere, negating the effect of the trough. The exception to this rule being the implementation of global or almost-global NPIs, which would synchronize the troughs, similar to what we considered in figure 5. If such troughs are sufficiently deep and synchronous, previously widespread pathogen lineages may die out. An example of this may have been observed, with the probable extinction of the Yamagata lineage of influenza B virus [77].

## 5. Limitations

Our transmission model assumes mean-field behaviour, relying on a homogeneous mixing assumption and neglects any person-to-person heterogeneities in, for example, susceptibility and infectiousness. While these are often reasonably accurate assumptions [78,79], it has been shown that departures from uniform infectiousness can have profound implications for epidemic dynamics, especially when social contact networks are restricted (such as during lockdowns), representing a departure from homogeneous mixing [80–82]. Evolutionary models have suggested that the two may even be coupled by evolutionary pressures, with NPIs affecting the evolutionarily favoured transmission patterns of pathogens [83]. In the bacteriophage experiments and associated model of [12], it was demonstrated that heterogeneity in strain-specific immunity across a host population attenuates the emergence risk, another type of heterogeneity that our current analysis does not address.

While we have included both imperfectly infection-blocking and finite-duration ( ‘waning ’) immunity in the model presented here, a more detailed model of partial immunity is likely to lead to further insights. In [84], the authors use an antibody titer model to describe partial immunity, a feature that could possibly be included in a more elaborate model of the net adaptation rate.

Diversity in vaccine-induced immune responses constitutes another source of heterogeneity that we did not include, which may affect the establishment probability of escape variants. This phenomenon was studied by Bouman *et al*. [85], who introduced the concept of *escape depth* to describe the fraction of escape mutants likely to spread in a vaccinated population.

Finally, and perhaps most importantly, our highly idealized model does not include any detailed antigenic fitness landscape. Studies on the relation between antigenic and genomic distance suggest a correlation between the two [86,87], while antigenic cartography indicates that variants are typically well separated in antigenic space [88–91]. Both of these findings challenge the memory-less constant-rate process assumption we make in this article. We anticipate that it may be possible to refine the model by more explicitly modelling genomic and antigenic distances and incorporating data on typical relations between the two.

## 6. Concluding remarks

In this study, we have proposed a framework to explore the dynamics of viral escape variant emergence, in which a population-scale phylodynamic curve emerges dynamically, encapsulating the nonlinear relationship between population immunity and viral adaptation. The framework contributes to a broadening understanding of the conditions under which viral immune escape variants are most likely to emerge, providing valuable insights for public health interventions and vaccine strategies.

We have shown that, far from being a static quantity, the same mathematical expression of the net rate of adaptation that gives rise to the phylodynamic curve can straightforwardly be coupled to dynamical transmission models. This then allows evaluation of the risk of viral escape variant emergence in highly dynamical settings, including during public health interventions and seasonal variation.

Aside from the emergence of the nonlinear immunity/escape risk relation on the basis of a few simple assumptions, our main findings include:

— ‘Leaky’ (partially susceptibility-reducing) immunity gives rise to a non-monotonic immunity/escape risk relation, but purely waning all-or-nothing immunity does not.
— If immunity is leaky, the presence of waning does not substantially change the immunity/escape risk relation.
— In seasonally driven outbreaks, the bulk of the escape risk occurs in the initial stages of each outbreak. If case imports from regions with year-round circulation occur, the bulk of the escape risk may be shifted to the low-incidence trough period.
— NPIs may strongly reduce the risk of immune escape if uniformly applied across regions. Local NPIs may be much less effective in this respect if adjacent regions have continued circulation.
— *Polymicrobial effects*. Lifting of NPIs targeted against one pathogen may increase the escape risk in other pathogens.
— Explicit stochastic simulations (as well as theoretical arguments) justify lineage survival probability approximations made in the analytical formulation.

We stress that the expression given for the net adaptation rate can be applied to *empirical time series*. The equation involves the population fractions of susceptible, infected and recovered individuals, and while we have used dynamical models to supply these quantities, nothing prevents them from being supplied by empirical observations. This, however, does hinge on relatively high-fidelity susceptible reconstruction being performed, including the effects of multiple variant exposures and vaccinations where relevant, as well as estimation of the (time-varying) degree of under-ascertainment. While this represents a substantial effort, it is our hope that the framework will be applied to empirical time series in the future.

To effectively apply our model framework to real-world pathogens, several types of data will thus be essential. Accurate data on recovery rates, transmission rates and infection prevalence are of course crucial for applying the model in any given scenario. Perhaps more crucially, the model relies on good estimates of the strength and duration of (cross-) immunity and the ability to perform (multi-variant) susceptible reconstruction and thus benefits from input from serological studies.

The framework we propose offers a foundation for emergence risk evaluation. By applying the model across various pathogens, we may gain insights into the differing propensities of viruses to develop immune escape variants. This comparative approach is likely to elucidate the factors that make certain pathogens more susceptible to rapid evolution, thereby informing targeted surveillance efforts and enhancing intervention strategies. Moreover, our model can serve as a tool for optimizing vaccine design and distribution. By simulating different vaccination strategies, the framework may help identify the conditions that minimize the net adaptation rate.

Finally, the evolutionary consequences of the COVID-19 NPIs across circulating pathogens are still being examined and evaluated. Our proposed framework can form part of a theoretical basis for these evaluations, providing insights into how interventions may influence the evolutionary dynamics of multiple pathogens.

## Data Availability

The program code developed for this study is available on Zenodo [92]. Electronic supplementary material is available online [93].
